# Supplementary material for: Are carbon emissions trading and green financial instruments synergistic? -Comprehensive quantitative research based on content analysis
Source: PLoS One. 2024 Mar 7;19(3):e0298601. doi: 10.1371/journal.pone.0298601 (PMC10919601; doi:10.1371/journal.pone.0298601)
Supplement: S1 Data — (ZIP) [file pone.0298601.s001.zip › Data/地级市Z_F工作报告.zip!/║╙▒▒/╠╞╔╜╩╨/└┤╫╘╣1⁄2╓┌║┼í╛┬φ┐╦╩2╛▌═°í┐_2009.docx]

一、2008年政府工作回顾

刚刚过去的2008年，是很不寻常、很不平凡的一年，也是本届政府履职的第一年。面对宏观经济形势的深刻变化和各种严峻挑战，全市人民在省委、省政府和市委的正确领导下，认真贯彻落实中央、省一系列决策部署，以科学发展示范区建设为总揽，以深入学习实践科学发展观活动为动力，紧紧围绕“开放创新、富民强市，把新唐山建成科学发展示范区、建成人民群众幸福之都”的总战略和总目标，抢抓机遇，开拓创新，拼搏进取，较好地完成了市十三届人大一次会议确定的各项目标任务，科学发展示范区建设迈出了新的步伐。

——国民经济保持平稳较快发展。预计全市完成地区生产总值3560亿元，增长13%;全部财政收入405.8亿元，其中地方一般预算收入146.6亿元，分别增长22.7%和23%。全年粮食总产287.9万吨，增长0.5%，连续五年实现增产;规模以上工业增加值和利税分别增长14.5%和14%;服务业增速达到15.5%。项目支撑作用显著增强，全社会固定资产投资完成1361.3亿元，增长31.3%，其中市级以上重点项目完成投资476亿元，350公里高速动车组、蓝欣镀膜玻璃一期等一批重点项目完工投产。节能减排取得重要进展，全市单位生产总值能耗同比下降5.27%，二氧化硫、化学需氧量排放量分别削减6.5%和11%，可以完成省下达的目标任务。

——唐山湾“四点一带”开发建设势头强劲。“四点一带”地区经济总量和投资规模分别占全市的30.6%和47.7%，其中曹妃甸工业区完成投资327亿元，增长36.6%。唐山湾“四点一带”开发建设规划体系进一步完善。曹妃甸新区获省委、省政府批准正式设立，进入产业聚集加速发展的新阶段。首钢京唐钢铁厂一期工程第一部分基本建成，中海油服装备制造基地等一批重点产业项目开工建设;曹妃甸港区30万吨级原油码头和煤炭码头一期工程完工，LNG码头完成选址，京唐港区3000万吨专业煤炭泊位竣工。唐山港货物吞吐量突破亿吨大关，跨入亿吨大港行列。唐曹高速建成通车，津秦铁路客运专线、承唐高速二期等工程开工建设，供水、供电等配套设施日臻完善，填海造地形成陆域120平方公里。“四点一带”已经成为拉动唐山跨越发展的龙头。

——城镇面貌三年大变样成效显著。全市投入城镇建设资金400多亿元，为历年来投入最多的一年，也是城市面貌发生变化最大的一年。新一轮城市总体规划修编工作完成。城市“四大功能区”规划建设全面启动，曹妃甸新城总体规划编制完成，起步区完成造地5平方公里，滨海大道开工建设;凤凰新城一批市政基础设施、公建及商业住宅项目开工建设;南湖生态城拓展湖面8平方公里，地震遗址公园、垃圾山封山绿化、环湖景观大道等工程主体完工;空港城概念性总体规划完成。拆违、拆迁830万平方米，累计拆除1130万平方米，占全市建成区建筑总面积的11.3%。震后危旧平房改造加速推进，新开工安置住房155万平方米，累计达到235万平方米。北新道等12条城市道路综合改造完工，长青楼区域改造等旧城改造项目开工，16个城中村改造启动实施，既有居住建筑节能改造完工121万平方米，城市绿、美、亮、净环境综合整治成效明显，完成建筑亮化550栋。组建了城市管理行政执法局，推行城市“网格化”管理，城市管理水平有了新的提升。军民两用机场已经国务院、中央军委批复立项。县城和小城镇建设迈出新步伐。

——改革开放扎实推进。国有企业改革、国有资产管理体制和财政体制、投融资体制等各项改革取得新进展，渤海、长城两大地方钢铁集团正式挂牌，整合重组和新建了12家国有投资公司。民营经济快速发展，增加值占全市生产总值比重达到61.8%。全市实际利用外资8.6亿美元,增长29.4%;进出口总额90亿美元,其中出口50亿美元，分别增长73.4%和76.7%。与日本住友、中海油、中材集团等一批国内外大公司、大集团的战略合作迈出实质性步伐。成功举办了唐山·曹妃甸临港产业国际合作会议、第十一届唐山中国陶瓷博览会，取得丰硕成果。我市荣获中国改革开放三十年优秀集体称号。

——新农村建设迈出新步伐。全年投入“三农”资金22亿元，增长22%。现代农业发展步伐加快，乳业、瘦肉型猪、果菜、板栗、花生、水产品等六大龙型经济健康发展，农业产业化经营率达到61%。农民增收渠道拓宽，非农收入占农民人均纯收入的68%。新建改建农村公路963公里，新建沼气池10.3万户，解决了2450个村、217.7万人的饮水安全问题。新创建文明生态村454个，累计达到总村数的61%。科学发展示范村创建活动深入开展，90%的村建成村民中心，完成283个村、3.1万户的“平改坡”工程，以“六个一”新农居改造为代表的科学发展模式试验示范工作收到实效，农村面貌发生可喜变化。

——社会事业发展取得新进步。全市财政投入社会事业资金达114亿元，增长41.4%。科技创新能力进一步增强，取得国内先进水平及以上科技成果306项，中科院唐山高新技术研究与转化中心正式成立。免除10.6万名公办普通高中公助生基本学费，免除义务教育阶段农村学生和城市低保家庭学生教科书费，改造农村陈旧校舍10.3万平方米;高等教育发展规划通过初步论证，河北理工大学北校区、华北煤炭医学院新校区一期工程完工。城市社区卫生服务机构达到130个，社区卫生服务人口覆盖率达到100%;新建农村卫生室748个，累计达到5556个，实现“一村一室”目标。文化事业和文化产业蓬勃发展，《人影》、《香妃与乾隆》等优秀作品获国家级大奖，开滦国家矿山公园等一批文化产业项目顺利实施，成功举办了第六届中国评剧艺术节。全民健身运动广泛开展，新建20个社区健身苑、100个农民体育健身工程。计划生育、民族宗教、广播电视、外事侨务、爱国卫生、防震减灾、妇女儿童、老龄、气象、档案、人防、“双拥”、民兵预备役和国防动员等各项事业全面发展。深入开展创建全国文明城市活动，我市首次进入全国文明城市候选城市。

——人民群众生活持续改善。预计城镇居民人均可支配收入和农民人均纯收入分别达到16382元和6625元，分别增长15.1%和13.7%。城镇新增就业6.8万人，城镇登记失业率控制在4.4%。城镇职工养老、失业、工伤保险进一步完善，在全省率先实现“全民医保”制度全覆盖，农村新型养老保险试点扩大到7个县(市)区，市区低保标准由每人每月225元提高到270元、农村低保标准由每人每年1000元提高到1200元。开工建设经济适用住房68.2万平方米、廉租房5.2万平方米。深刻汲取三鹿奶粉事件和我市安全生产事故教训，深入组织安全生产大检查，集中开展“打击非法盗采国有矿产资源专项行动”、“食品安全检查和专项整治”以及“矿山钢铁焦化等行业安全专项整治”，努力维护人民群众生命财产安全。我市被评为2008年中国最具幸福感城市。

各位代表，在宏观经济形势复杂多变，尤其是去年下半年以来国际金融危机蔓延、宏观经济环境面临诸多困难的严峻形势下，我市经济社会发展取得这样的成绩来之不易。一年来，我们认真贯彻落实科学发展观，积极应对各种严峻挑战，抓重点、攻难点、破瓶颈，着力发展经济，深化改革开放，保障和改善民生，促进社会和谐，付出了艰苦努力。

(一)认真开展学习贯彻十七大精神和深入学习实践科学发展观活动，进一步夯实建设科学发展示范区的思想基础和工作基础。按照市委统一部署，把学习贯彻十七大精神与开展深入学习实践科学发展观活动紧密结合起来，与建设科学发展示范区紧密结合起来，引导政府系统广大党员干部解放思想、更新观念、理清思路、强化措施，坚定不移地把十七大精神落到实处。围绕“党员干部受教育、科学发展上水平、人民群众得实惠”这个总要求，在各级政府机关扎实开展深入学习实践科学发展观试点工作各个阶段的活动，形成了科学发展的强烈共识，凝聚了科学发展的强大力量，科学发展成为全市上下的自觉行动。

(二)积极应对宏观经济形势带来的严峻挑战，力促经济平稳较快发展。针对国际金融危机快速蔓延、经济放缓的严峻形势，全面落实国家宏观调控政策，制定出台了《关于应对当前宏观经济形势实现又好又快发展的若干意见》和《关于促进全市经济平稳较快发展的实施意见》。特别是抢抓国家实施扩大内需政策的机遇，全力谋划和争取项目。全市共谋划重点推进项目1359个，总投资规模约1万亿元。采取联合审批形式，加快项目审批和前期工作进度，累计向国家上报重大项目61个，申请中央投资65.3亿元，其中35个项目已获得批准，利用中央投资3.74亿元。京唐城际高速铁路、曹妃甸中石化大型炼化一体化、曹妃甸矿石码头二期等一批重大项目取得突破性进展;加强与金融机构的对接与合作，争取建行、农行、国家开发银行授信额度1500亿元。及时有效的应对措施，使我市经济保持了平稳较快发展的良好态势。

(三)抓住事关唐山发展的战略重点全力加以推进，努力带动和活跃工作全局。着眼于加快资源型城市转型，积极推进城市功能转型、产业结构转型、企业和体制转型。尤其是围绕产业结构优化升级，出台了促进产业链经济发展的政策措施，精品钢材、基础能源、优质建材、装备制造和化工五大产业增加值占规模以上工业的80.3%，服务业发展高于GDP增速2.5个百分点，高新技术产业增加值增长20%。着眼于打造唐山跨越发展的战略支撑，制定实施了支持“四点一带”开发建设若干政策，举全市之力加快唐山湾开发建设步伐，使“四点一带”在启动之年取得重大进展。着眼于加快城乡一体化，坚持把“三农”问题作为重中之重，遵循城乡等值化发展理念，编制完成《城乡发展一体化战略规划》，扎实推进让农民富起来、农户暖起来、农村靓起来、农家乐起来、农村经济循环起来“五个起来”，全面落实农民工进城落户、农民进城就业、农民工子女就读、农民进城公共交通、农民进城就医报销“五个无障碍”，推动了新农村建设深入开展。

(四)集中力量打了五场攻坚战，着力破解影响和制约科学发展的突出问题。围绕破解生态环境脆弱的难题，开展了绿化唐山攻坚行动，实施城镇及周边绿化、通道绿化、村庄绿化、农田及“四荒”绿化、工业园区及企业绿化、矿山修复绿化等六大工程，完成造林绿化42.4万亩，相当于近3年的总和，森林覆盖率达到26.45%，提高2.1个百分点。围绕破解城市建设与经济发展不相适应的难题，开展了城乡建设改造和环境综合整治攻坚行动，强力实施“114421”工程，推动省委、省政府城镇面貌三年大变样工作部署的落实。围绕破解资源能源消耗高、环境压力大的难题，开展了安全生产节能减排持续攻坚行动，实施“双三十”和“10100”等七大工程，对钢铁等10大重点领域的4591家企业进行综合整治，关闭取缔“十小”企业236家，停产整顿高耗能、高污染企业267家;组织开展了淘汰水泥机立窑和中空窑集中行动，拆除丰董线等区域的水泥窑34座，淘汰落后产能370.2万吨。围绕破解发展后劲不足、发展方式转变不快的难题，开展了重点产业项目建设攻坚行动，筛选50个重大产业项目集中力量实施攻坚，其中三友集团盐化工产业链等23个项目开工。围绕破解发展成果普惠性不强的难题，开展了改善民生攻坚行动，投入财政资金30亿元，集中为群众办了20件实事，并已全部完成，使改革发展的成果更多的普惠于广大人民群众。

(五)全力做好奥运安保和维护稳定工作，为改革发展营造良好环境。北京奥运会期间，围绕保障奥运绝对安全，把奥运安保工作作为一个时期压倒一切的政治任务，以最高的标准、最严的要求、最大的努力，坚决打好奥运安保这场硬仗。圆满完成了奥运火炬在我市的传递活动和北京奥运安保各项任务，全市人民受到一次深刻的爱国主义教育，进一步激发了建设新唐山的政治热情。高度重视群众信访工作，开展市县乡村四级大接访活动，化解了一大批多年沉积下来的信访突出问题。强化社会治安综合治理，积极探索维护稳定综合调控机制，保持了全市政治安定、社会稳定的良好局面。

(六)注重体制机制创新和政府职能转变，不断提高行政服务水平。围绕激发政府管理活力、市场主体活力和金融活力，加快政府职能转变，研究制定了向县区下放权力、加强要素建设和优化配置、促进循环经济发展等多项政策措施，着力破除体制机制障碍，创优发展环境。认真贯彻落实《行政许可法》和《公务员法》，加强政府法制建设，推进依法行政。深入开展“市民献计献策月”活动，倾听民声、了解民情、集中民智，使政府工作更加符合人民群众的意愿和要求。扎实开展“效率年”活动，推行“电子政务”、网上审批、超时默许、零成本注册，行政许可和非行政许可类审批项目分别由523项、463项减少到190项和102项。加强廉政建设，推行行政权力公开透明运行。自觉接受人民代表大会及其常委会的依法监督，重视发挥人民政协的政治协商、民主监督和参政议政作用，承办市以上人大代表批评意见、建议和政协提案767件，办结率100%，促进了政府工作水平的提高。

需要指出的是，四川汶川特大地震发生后，按照市委的决策部署，倾力做好支援四川灾区抗震救灾工作。全市干部群众捐款捐物合计3.38亿元，到灾区抢险救灾的志愿者达1700多人。全市上下众志成城支援灾区抗震救灾的行动，得到了党中央国务院和省委省政府的高度评价，受到了灾区干部群众和社会各界的广泛赞誉，唐山“爱心之城”的城市名片更加响亮。特别是“感恩、博爱、开放、超越”的新唐山人文精神，得到了空前的弘扬和升华。

各位代表，通过全市上下的共同努力，我们克服了前进道路上的各种困难，实现了新的发展，为加快建设科学发展示范区和人民群众幸福之都奠定了更加坚实的基础。这是党中央国务院、省委省政府和市委正确领导的结果，是全市人民同心同德、锐意进取、艰苦奋斗的结果。在此，我代表市政府，向全市工人、农民、知识分子、各级干部和广大建设者，向人大代表、政协委员，向各民主党派、工商联、无党派人士和人民团体，向驻唐部队、武警官兵、公安政法干警以及关心支持唐山发展的各界朋友，表示崇高的敬意和衷心的感谢!

总结过去的一年，我们也清醒地认识到，我市经济社会发展中还存在许多亟待解决的问题，前进道路上还面临许多新的挑战。主要是：在当前复杂的宏观经济形势下，经济发展面临的不确定因素增多，经济下行压力加大，部分企业生产经营困难、效益下滑、开工不足，就业形势严峻;经济结构性矛盾仍比较突出，加快发展方式转变的任务还很重，节能减排、安全生产任务十分艰巨;支撑科学发展的制度体系有待进一步创新和完善，深化改革的任务还十分紧迫;改善民生的措施需要进一步强化，社会保障、人居环境等一些关系群众切身利益的问题还没有得到更好地解决;政府自身建设和管理需要进一步加强，一些政府工作人员服务意识不强、官僚主义、办事效率低下等问题还比较突出，以权谋私等腐败现象仍然存在。对这些问题，我们要引起高度重视，在今后工作中认真加以解决。

二、2009年工作总体要求和奋斗目标

今年是建国六十周年，也是推进“十一五”规划顺利实施的关键一年。做好今年工作，对于维护改革发展稳定大局，实现“十一五”规划确定的目标任务，推进新唐山科学发展示范区建设具有重要意义。当前，国际金融危机仍在扩散和蔓延，对实体经济影响正在进一步加深，国内经济运行有可能面临更加困难的局面。这些都给我市经济发展带来了严峻挑战。对此，我们一定要切实增强忧患意识、危机意识，有清醒的认识和足够的准备。同时，我们必须充分认识到，我国经济发展的基本面和长期趋势没有改变，我们遇到的困难和挑战只是前进中的问题。就我市来讲，加快发展面临许多有利条件。应该看到，经过改革开放30年来的快速发展，我市综合经济实力显著增强，已成为河北第一经济强市和省域中心城市，抵御风险、应对挑战能力显著提升;应该看到，唐山所处的黄金发展期仍然存在，尤其是国际金融危机引发的全球经济调整、国家实施积极的财政政策和适度宽松的货币政策，又为唐山带来了加快科学发展的新机遇、实现跨越发展的新机遇、扩大开放的新机遇、改善民生的新机遇;应该看到，随着唐山湾“四点一带”、城市“四大功能区”开发建设等重大战略部署的实施，我市产业优势、港口优势、资源优势、成本优势、环境优势正在集中凸显，为唐山吸纳国内外优势生产要素以及在新一轮调整发展中抢占先机创造了有利条件;尤其应该看到，在困难和挑战面前，全市广大干部群众思想高度统一、目标高度一致、建设科学发展示范区的热情空前高涨，这是我们战胜一切艰难险阻的根本保证。只要我们审时度势，主动应对，完全有条件变压力为动力、化挑战为机遇，开创唐山科学发展新局面。

前不久召开的市委八届五次全会，基于对当前宏观形势的科学判断，基于对科学发展示范区建设的实践探索，在坚持市委八届三次、四次全会以来形成的一系列发展思路的基础上，对今年工作做出了战略部署，为加快科学发展示范区建设进一步指明了方向。我们一定要按照市委工作部署，抢抓机遇，坚定信心，攻坚克难，埋头苦干，努力把唐山改革发展推向一个新境界。

2009年全市国民经济和社会发展的总体要求是：全面贯彻党的十七大、十七届三中全会和省委七届四次全会、市委八届五次全会精神，以邓小平理论和“三个代表”重要思想为指导，更加自觉地深入贯彻落实科学发展观，继续以科学发展示范区建设为总揽，以保持经济平稳较快发展为首要任务，把加大投资、扩大内需作为根本途径，把加快发展方式转变和结构调整作为主攻方向，把深化改革、扩大开放作为强大动力，把改善民生作为出发点和落脚点，深入推进新型工业化、新型城镇化、城乡等值化、社会管理科学化，朝着建设科学发展示范区、建设人民群众幸福之都的宏伟目标加速前进，以优异成绩向建国六十周年献礼!

全市经济社会发展主要预期目标是：地区生产总值增长11%以上;全部财政收入增长12%;城镇居民人均可支配收入增长10%以上，农民人均纯收入增长8%以上;全社会固定资产投资增长30%以上;单位生产总值能耗下降5.28%以上，化学需氧量排放量减少6%，二氧化硫排放量减少5%;城镇登记失业率控制在4.4%以内;人口自然增长率控制在4.98‰。上述发展目标，是在全面分析形势的基础上确定的，与我市“十一五”规划相衔接，需要全市上下团结奋斗、共同努力去实现，并力争完成得更好一些。

做好今年政府工作，实现今年经济社会发展目标，必须按照科学发展观的要求，坚持统筹兼顾，正确处理好“四个关系”：一是正确处理当前与长远的关系。立足当前保增长，着眼长远增后劲，既把保持经济平稳较快发展作为当前的首要任务，又多做打基础、利长远的事，谋划实施一批事关唐山未来发展的重大支撑项目和基础性工程，为实现可持续发展奠定坚实基础。二是正确处理好与快的关系。越是在加大力度保增长的时候，越要重视质量和效益，加快结构调整和发展方式转变，把实现保增长目标建立在提高质量、优化结构、增加效益、降低消耗、保护环境的基础之上。三是正确处理政府与市场的关系。既要注重发挥市场在资源配置中的基础性作用，坚持按市场规律办事，又要主动作为，积极发挥政府在促进引导、支持服务、规范管理等方面的作用，激发各方面加快发展的积极性。四是正确处理发展与稳定的关系。发展是第一要务，稳定是第一责任。更加重视和改善民生，让发展成果更多、更广泛的惠及人民群众。把握好各项改革的推进力度,区分轻重缓急,做到有序推进;把握好社会各阶层的承受能力,各项措施的出台兼顾不同利益群体的意愿和需求,在维护社会稳定中推进改革发展，通过改革发展促进社会稳定。

三、2009年重点工作任务

市委八届五次全会确定，今年在全市集中力量实施“五项攻坚行动”、深入推进“八大工程”，以此推动经济社会又好又快发展，把科学发展示范区建设进一步引向深入。我们要围绕落实市委战略部署，把握关键，强化措施，全力推进十项重点工作：

(一)以千个项目保增长调结构攻坚行动为重点，促进经济平稳较快发展。发展是解决一切问题的关键。抓发展，核心是抓项目。抓住国家实施扩大内需、促进经济增长的政策机遇，深入实施千个项目保增长、调结构攻坚行动，努力增强经济发展后劲和可持续发展能力。

加大项目谋划推进力度。明确主攻方向，力促结构调整，围绕基础设施建设、产业发展、新农村建设、拉动消费和改善民生等重点领域，加大项目谋划和跑办力度，力争更多的项目纳入中央和省的盘子，确保早开工、早落地、早见效。全市重点推进1000个项目。着力推进一批基础设施项目，加快唐山机场、京唐港通用杂货泊位、承唐高速二期、津秦客运专线等项目建设，做好京唐城际高速铁路、蒙冀铁路张曹线等一批基础设施项目前期工作，力争早日立项开工;着力推进装备制造、化工、高新技术、现代服务业等一批重大产业支撑项目，重点抓好高速机车装备制造基地、住友装备制造基地、曹妃甸装备制造基地、曹妃甸石油炼化基地、乐亭煤化工基地、南堡盐化工基地等重大项目的实施，谋划建设若干个高新技术产业园区和信息产业基地，推进一批重大现代服务业项目建设;着力推进一批新农村建设和民生改善项目，重点加快保障性住房、卫生文化基础设施和新农村社会事业发展等项目建设。在项目谋划建设中，坚持扩大投资规模与优化投资结构并举，严格执行国家产业政策，严格控制高耗能、高污染和产能过剩行业投资，坚决防止低水平重复建设和盲目扩张。切实加强投资项目和资金监管，提高投资质量和效益。

充分发挥企业主体作用。鼓励支持大型骨干企业调结构、上项目、求发展，争当行业排头兵，进一步发挥其在全市发展中的支撑带动作用。高度重视并支持中小企业、民营企业做优做强，加大对成长型企业和特色产业集群扶持力度，培育一批新的市场主体，建设一批新的中小项目，充分发挥其在促进发展、吸纳就业、改善民生等方面的重要作用。进一步激发民营经济发展活力，切实加强信用担保体系、创业辅导体系、技术创新服务体系及人才培训体系和信用评价体系建设，形成活力迸发、全民创业的生动局面。当前，部分企业生产经营出现一些困难，要给予企业更多的支持和帮助，为企业创造更好的发展环境。加强对企业的分类指导和服务，对市场前景好、需求旺的企业，全力保障各类生产要素供应，最大限度的促其增产增效;对平、滞销的企业，有针对性地帮助其调整结构、开拓市场，力争保持正常生产;对设备、工艺、技术落后，产品没有前景的企业，积极推进兼并重组，向市场前景好、产业链条长、抗风险能力强的大企业、大集团靠拢，实现共同发展。加强企业诚信教育，引导企业打造诚信品牌。

着力破解资金、土地等瓶颈制约。抓住国家实施适度宽松的货币政策机遇，进一步推进银企合作，充分发挥商业银行融资主渠道作用;加快唐山商业银行与北京银行战略合作，力争民生银行、招商银行等股份制银行早日在唐设立分支机构;大力发展资本市场，鼓励企业上市和发行公司债券，加快发展钢铁期货交易，积极探索发展信托投资公司、私募股权投资基金;积极规范发展小额贷款公司等新型金融组织，为项目建设和企业发展提供更加有力的金融支撑。在积极争取国家和省更多建设用地支持的同时，充分挖掘存量土地潜力，搞好土地修复置换，用好沿海滩涂荒地，提高土地利用率和集约化水平。

(二)以曹妃甸新区为龙头，全面加速唐山湾“四点一带”开发建设。唐山湾“四点一带”是唐山科学发展的强大引擎和增长极。以更大的气魄、更大的力度推进沿海地区开发开放，掀起开发建设新高潮。“四点一带”地区生产总值和财政收入增速高于全市增速两个百分点;曹妃甸新区力争完成投资1000亿元。

加快完善规划体系。加快“四点一带”区域发展规划及产业、交通、环境等专项规划的调整，上半年编制完成曹妃甸新区、乐亭新区、丰南沿海工业区和芦汉经济技术开发区发展规划、环境规划和各产业园区的控制性详规，年内完成唐山港总体规划修编。加快海洋功能区划调整修编工作，保障项目建设的用海需求。

加快推进产业聚集。坚持把产业聚集放在突出位置，加快引进一批战略投资者，建设一批大项目，打造沿海产业集群带。曹妃甸新区，围绕发展精品钢铁、装备制造、石化、海洋化工和现代物流、高新技术产业，重点抓好中海油服装备制造、华电集团临港装备制造产业基地等项目建设，积极推进中远集团修造船、中石化大型石油炼化一体化等重大产业项目前期工作，力争取得突破、早日开工建设。乐亭新区，围绕发展精品钢材、装备制造，煤化工等主导产业，加快唐齿变速器、嘉恒重工等在建项目建设，做好旭阳煤化工、中润煤化工二期、京唐港区保税仓库等一批项目前期工作，力争早日开工。丰南沿海工业区，围绕发展装备制造、新型建材等主导产业，重点抓好国丰冷轧镀锌板等项目建设，积极推进冶金矿山与长沙中联重科合作建设冶金矿山装备制造生产基地等项目。芦汉经济技术开发区，围绕发展高新技术产业、五金制品等优势产业，重点抓好文化创意、动漫、信息服务与软件外包、信息安全产业园区建设，打造北方信息产业基地;抓好螺旋焊管、泰美自行车、镀膜玻璃二期等重点项目建设。

加快基础设施建设。抢抓国家扩大内需的机遇，强力推进港口、公路、铁路等综合交通体系建设。加快曹妃甸港区通用码头、滨海大道等重大项目建设进度，力争早日完工;积极做好曹妃甸港区矿石码头二期、LNG码头和京唐港区10万吨级航道工程等项目前期工作，尽快全面开工建设。同时，加快供电、供水、造地等配套工程建设进度，开工建设海水淡化项目。

加快体制机制创新。进一步完善管理体制，加快乐亭新区、芦汉经济技术开发区管理体制改革。做好曹妃甸新区综合改革试验区申报工作，争取国家政策支持。创新投融资体制，设立曹妃甸产业投资基金，尽快运作曹妃甸投资控股公司，推进唐山港股份有限公司上市融资。制定人才激励政策，不拘一格引进高层次人才，真正形成人才高地。

在加快唐山湾“四点一带”开发建设的同时，统筹推进北部山前经济带和城市带发展。充分发挥其资源、区位、产业等优势，加强基础设施建设，改造提升钢铁、建材、机械制造等传统产业，优先发展装备制造业、高新技术产业和现代服务业，培育壮大旅游、农产品加工、商贸流通等特色产业，加快推进资源型经济转型，使北部山前城市带与沿海“四点一带”形成功能互补、政策互动、产业互融、设施互联、互为支撑的一体化发展新格局。

(三)以现代产业体系建设为核心，加快资源型城市转型步伐。把资源型城市转型作为科学发展示范区建设的战略任务，牢牢抓住产业转型这个核心，以发展产业链经济为重点，以节能减排为抓手，以园区经济为载体，以科技创新为动力，加快产业结构调整和发展方式转变，努力推动唐山这座资源型城市焕发出新的活力。

积极发展产业链经济。按照新型工业化要求，继续培育壮大精品钢材、装备制造、化工、现代农业、现代服务业、高新技术和环保七大主导产业链。重点组织实施好100项重大产业链支撑项目，年内完成投资450亿元以上。特别是加快精品钢材、现代装备制造和化工产业发展，尽快形成“三足鼎立”的产业格局。钢铁产业，积极推动首钢京唐钢铁厂尽早正式生产，支持长城、渤海等大型优势钢铁集团壮大发展，进一步加快整合重组和淘汰落后产能步伐，推进装备大型化和高科技化，提高产业集约化水平;加快钢铁产品结构调整，向中高端、深加工迈进，全市精品钢比重达到55%。装备制造业，依托住友重工、轨道客车、曹妃甸装备制造产业园等骨干企业和园区，推进工程机械、水泥及冶金工程成套设备、临港重型装备制造等重点项目建设，加快打造现代装备制造产业基地。化工产业，在继续发展煤化工、盐化工的同时，把1000万吨炼油/100万吨乙烯大型石油炼化一体化作为重中之重，力求突破。加快用高新技术改造提升传统产业，打造高新技术产业集群，实施企业重点技术创新和高新技术产业项目50项。以推进信息化和工业化融合为重点，加快企业信息化建设。高度重视现代服务业发展，全面落实鼓励支持政策，在提升商贸流通等传统服务业同时，加快发展现代物流、金融服务、商务服务等生产性服务业，积极发展旅游、商务休闲、文化创意、社区服务等新兴服务业，重点抓好远大物流综合服务平台、唐山湾“三岛”旅游等100个重点服务业项目。推进大型工业企业生产性服务业的剥离工作。

强力推进节能减排。坚持把节能减排作为推进资源型城市转型和发展方式转变的重要抓手，巩固和扩大攻坚成果，强化工作措施，务求取得新成效。严格落实节能减排责任制。进一步加强对纳入省“双三十”和我市“10100”工程县(市)区、企业的监管考核，对节能减排工作进展缓慢、逾期完不成目标任务的地方和企业严格执行问责制和“一票否决制”。强化对高耗能、高排放企业监管，实现重点企业在线监测全覆盖。加快推进节能减排技术改造。推广260项国家鼓励发展的资源节约综合利用新技术和50项国家重点节能技术，抓好50项节能项目和100项减排项目建设。加大水泥、钢铁等高污染、高耗能行业落后产能淘汰力度，建立健全落后装备、高耗能产业退出机制和政府支持节能减排鼓励机制。积极发展循环经济。按照“减量化、再利用、资源化”的原则，组织实施一批循环经济示范工程，加快曹妃甸国家循环经济示范区、司家营循环经济园区建设。注重运用经济手段推动节能减排，全面落实差别电价、阶梯水价政策。

加快发展园区经济。积极推进园区集约发展模式，重点支持曹妃甸工业区、高新技术产业园区、乐亭临港产业聚集区、开平现代装备制造产业聚集区等园区建设，加速形成各具特色的产业集群。重点支持各县(市)区加快园区建设，使之成为县域经济发展的重要支撑。

大力推进科技创新。加快建立以企业为主体、政产学研融合互动的科技创新体系，推进创新型城市建设。培育建设100家市级以上企业工程技术研发中心、行业重点实验室等科技研发机构，提高自主创新能力。抓好高速动车组自主研发基地和配套产业园区建设。围绕资源型城市转型、“四点一带”开发建设，实施15个重大科技创新和示范项目，转化150项重大专利技术。加强与国内外高等院校和科研院所的科技合作，充分发挥中科院唐山高新技术研究与转化中心作用，推进政产学研联合和高新技术成果转化。谋划建设唐山科技城和曹妃甸新城科教园区。

实施“人才强市”战略。创新人才政策机制，加大人才培养引进和使用工作力度，努力使人才队伍整体规模、综合素质和结构与唐山资源型城市转型和科学发展示范区建设相适应。

(四)以城镇面貌三年大变样攻坚行动为载体，加快生态城市建设。今年是推进城镇面貌三年大变样的关键一年。围绕建设现代化生态城市目标，全力实施城镇面貌三年大变样攻坚行动，加快城市建设改造步伐。全年城镇建设投资完成500亿元以上。

坚持以建为主、以提升品位为要，全力推进城市改造建设。突出抓好“四城一河”开发建设。把“四城一河”作为展示唐山崭新形象、拓展城市发展空间的核心工程、标志工程，科学规划，倾力推进。曹妃甸新城，全面开工建设，重点推进起步区土地整理、供电、供水等基础设施配套项目建设，启动曹妃甸大企业职工生活基地、曹妃甸新区行政中心等35个项目建设，实现城市与产业同步规划、同步实施、协调发展。凤凰新城，围绕建设一流区域商务中心、金融中心、总部基地、高新技术产业基地，加快基础设施建设和重点项目实施进度，尽快建成一批标志性建筑。南湖生态城，围绕建成一流的中心城区、生态城区和旅游度假胜地，加快景观绿化、路网体系等基础设施以及文化、娱乐、休闲旅游等项目建设。空港城，按照建成空中通道、空港物流区和高新技术产业园区的要求，抓紧编制详细规划和起步区控制性详规，尽快实现唐山机场民航通航，统筹加快空港物流业和高新技术产业发展。陡河、青龙河改造，重点完成引水工程、河道整治开挖和生态景观绿化，形成约54公里的环城水系;谋划启动商业、文化、旅游等项目，打造滨水生态景观带和服务业产业带。加快实施“三项改造”。继续把震后危旧平房改造作为“一号工程”，在已开工235万平方米安置住房的基础上，今年再开工240万平方米，力争市中心区危旧平房改造全部完成;加快市中心区城中村改造，全面启动39个改造项目;加快既有居住建筑综合节能改造步伐，年内完成500万平方米，到2011年全部完成2200万平方米改造任务。以老城改造和城市“四大功能区”开发建设为契机，加快建设一批富有时代气息、体现唐山特色、具有一流水准的城市标志性建筑，重点抓好万达广场、新华大厦、硅谷科技城等项目建设。全面实施县城扩容提升工程。完成县城规划建设用地控制性详规覆盖，力争经过几年努力，每个县城建成区在现有基础上拓展10平方公里以上、扩容10万人。加快县(市)城基础设施建设，年内每个县城至少建成一条景观大道、一条示范街和2-3个标志性建筑。

全面提升城市规划、建设、管理水平。按照生态理念高起点搞好城市规划。坚持城市建设规划第一、规划工作生态优先的原则，把生态理念和绿色元素融入“四城一河”开发建设、老城区改造和各县城以及乡村建设发展各项规划，使生态文明建设贯穿新型城镇化的全过程。引进国内外一流的设计机构参与城市规划，提升规划设计水平。运用市场手段筹集建设资金。充分发挥财政资金引导作用，鼓励民间资本、外商投资进入城市开发建设领域，积极引进战略投资者参与城市建设改造。推行BOT、BT等先进的经营运作模式，实行财政投资建设项目代建制，推进城市资源资本化。充分发挥各城市建设投资公司投融资平台作用，拓宽融资渠道，提高融资能力。规范房地产市场秩序，优化房地产发展环境。推进城市精细化管理。深入推行城市管理综合执法，加快城乡一体化管理服务信息系统建设，实现城乡数字网格化、精细化管理全覆盖。继续深入实施“绿、美、亮、净”工程，加强环境综合整治，进一步提升城市形象。

(五)以科学发展示范村创建攻坚行动为抓手，全面推进农村改革发展。创建科学发展示范村，是落实党的十七届三中全会精神的实际行动，是建设科学发展示范区的内在要求，是改善民生的重要举措。坚持从实际出发，实施科学发展示范村创建攻坚行动，努力把新农村建设引向深入。

在科学发展示范村创建上，以制定一个好规划、发展一个好产业、探索一个好模式、完善一套好制度、建设一个好班子为基本任务，尊重群众意愿，选取不同类型的若干村，加强引导，典型示范，全力推进。一是大力推进新民居建设，全市重点抓100个新民居整体新建示范村、1万个新民居示范户，按照冀东特点、唐山特色规划新建改建村庄和民宅，积极探索集约节约利用土地、集约利用宅基地新模式;二是大力推进旧民居改造，重点抓好200个以住宅主体结构改造为主要内容的旧民居改造示范村，500个以博士灶、吊炕、太阳能利用、改水改厕等为主要内容的生活设施改造示范村，促进“六个一”模式的普及推广;三是大力推进中心镇连片创建，选择若干示范镇进行村庄连片创建，每个县(市)区重点规划培育1-2个规模在5万人左右的中心镇，达到科学发展示范镇水平。

在农村改革发展上，立足农民持续增收、农业增产增效和农村和谐稳定，重点抓好三个方面：一是加快农村体制机制创新。创新农村经营体制和机制，坚持以家庭承包经营为基础、统分结合的农村双层经营体制，积极探索集体土地多种有效实现形式;以股份合作为主要形式，鼓励支持农民发展各类合作社和专业合作经济组织。创新农村土地管理和流转机制，成立市县乡三级土地承包经营权流转交易中心(站)。创新农村现代金融机制，鼓励创建村镇银行、各类小额贷款公司，到2010年每个县(市)区至少设立一家村镇银行和小额贷款担保公司。创新乡村社会管理机制，深化乡镇机构改革，强化乡镇政府社会管理和公共服务职能。二是加快发展现代农业。稳定粮食生产，保障主要农产品有效供给。加大农业投入力度，加强农田水利基础设施建设，推进平原洼地治理，提高农业机械化水平。按照高产、优质、高效、生态、安全的要求，加快农业结构调整，推进农业产业化经营，抓好绿色奶源生产加工基地、瘦肉型猪生产加工良繁基地等特色农产品基地建设，全市销售收入超亿元的龙头企业达到28家，农业产业化经营率达到63%。大力培育农业品牌，打造集约高效型、循环生态型、外向型农业和订单农业。实施振兴奶业计划,做大做强唐山奶业自主品牌。完善现代农业综合服务体系，加强基层农业信息、技术推广、优良品种、农资农机、生产技术等综合服务。全市建设基层农技推广区域综合站26个。三是加快农村社会事业发展。积极发展农村教育、文化、卫生等各项社会事业，健全完善农村养老、医疗等社会保障体系。加强农村生态环境建设，改善农民居住和生活条件。实施乡村绿化工程，由县(市)区免费为每个农户提供10棵树苗。落实“农民素质提升计划”，建立农民培训长效机制，培育有文化、懂技术、会经营的新型农民。

(六)以城乡等值化为方向，加快推进城乡一体化进程。认真落实市委《关于推进农村改革发展加快实现城乡等值化的决定》，统筹推进城乡规划、建设、产业、公共服务、社会管理一体化，加快建立以工促农、以城带乡长效机制，尽快形成城乡经济社会一体化发展新格局。

促进城乡收入等值。通过产业发展、鼓励创业、落实惠农政策等多种渠道，千方百计增加农民收入，逐步缩小城乡收入差距。鼓励农民自主创业，以创业带动农民就业，以就业促进农民增收;大力扶持一批农业产业化龙头企业和农业产业化园区建设，加快发展农村二三产业，深入实施“阳光培训工程”，完善城乡劳动力市场服务体系，力争全市每个农户有一名劳动力在非农领域就业，增加农民工资性收入;推进农村土地的确权、登记、办证工作，用好农村宅基地等物权，通过宅基地置换、调整等方式，增加农民财产性收入;认真落实各项支农惠农政策，加大扶贫开发力度，确保低收入人口稳定脱贫。

促进城乡公共服务等值。加大对农村社会事业的投入，办一批惠农的好事、实事。年内改造陈旧校舍10万平方米，逐步推进“户均一名大学生”行动;进一步健全完善县、乡、村三级医疗卫生服务网络，乡镇卫生院标准化率达到100%;加强农村基层文化建设，年内支持建设50所乡镇综合文化站，繁荣发展农村文化。将城乡一体化管理服务信息系统延伸至乡、村，为农村、农民提供优质服务。鼓励农民进城，推进农村人口向城镇城区集中，统筹做好户籍管理、社会保障、住房安居、就业扶持、子女入学、宅基地流转等工作，解决农民进城后顾之忧，真正让农民进得来、留得住、过得好。

促进城乡社会保障等值。巩固和发展新型农村合作医疗保障制度，全市农民参合率巩固在94%以上;完善新型农村养老保险制度，进一步提高覆盖面和保障水平;加强“金保工程”建设，加快建立起覆盖全市乡镇和50%以上建制村的劳动保障信息网络;探索完善失地农民、失海渔民保障办法，逐步使农民享有与城镇居民等值的社会保障。

促进城乡生活便利程度等值。加强农村基础设施建设，实施农村公路联网工程，加快推进“道路入户”建设，积极发展农村沼气，强化村镇水资源管理。到2010年，全市农村基本实现户户通水泥路，适宜建沼气的村普及沼气池，基本普及自来水。加强农产品流通市场体系建设，继续抓好“万村千乡市场工程”、“农产品批发市场升级改造工程”，年内培育和发展规范化的农资连锁便民店1000家，农村日用消费品便民超市100家。

(七)以增强科学发展动力和活力为立足点，努力深化改革、扩大开放。坚定不移地推进改革开放，力争在重点领域和关键环节改革上取得新突破，在经济发展外向度上实现新提高，依靠改革开放增动力、添活力。

深入推进各项改革。加快国有企业改革。以产权制度改革为重点，年内全面完成市属国有企业改制。积极推进唐冶机械、唐山陶瓷等企业战略重组，大力引进战略投资者;全力组织实施市属困难企业改制攻坚，年内全部完成49户困难企业改制;把企业改制与“退二进三”相结合，加快冀东氯碱、启新水泥等企业搬迁改造步伐。同时，通过股份制、重组、拍卖等形式，加快供销社企业改革。加快行政管理体制改革。围绕转变政府职能，进一步完善行政管理体制，推进行政审批制度改革，减少审批层级和环节，完善行政审批“一站式服务”。按照国家、省统一部署，做好政府机构“大部制”改革工作。稳步推进事业单位改革。加快财政体制改革。推进增值税转型改革，落实促进经济增长的激励性财政政策。深化预算制度改革，建立财政支出责任体系。牢固树立科学理财和艰苦奋斗思想，进一步加强税源建设，调整优化支出结构，严格控制一般性支出，发挥财政资金最大效益。加快科技体制改革。深化科技计划管理体制、产学研协作创新体制、科技成果管理体制三项改革，用好科技专项经费，激发科技创新活力。加快教育体制改革。按照素质教育要求，探索建立更具活力的教育管理体制，启动实施义务教育学校绩效工资制;着眼于唐山湾“四点一带”开发建设对高素质技能人才的需要，整合教育资源，加快体制机制创新，建设一流高等职业技术学院。加快文化体制改革。加快经营性和公益性文化事业单位改革，健全文化产业多元化投入机制，使文化事业单位更加充满活力，使文化企业蓬勃发展。加快社会管理体制改革。切实抓好养老保险、医疗保险、户籍制度等一系列社会管理制度的改革。

全力推进双向开放。适应国际资本流动新趋势和国际市场新变化，引导全市上下进一步强化开放意识，营造全民促开放的浓厚氛围，以大开放促进大发展。加大招商引资力度。落实招商引资奖励政策，形成全民招商生动局面。改进招商方式，增强招商的针对性和实效性。办好科学发展(曹妃甸)论坛、曹妃甸临港产业国际合作会议、唐山中国陶瓷博览会，打造在国内外具有较强影响力的对外开放平台。积极推进签约项目的履约、落地和开工建设，重点抓好爱信二期增资、曹妃甸华润电厂等30个1000万美元以上利用外资项目和首钢装备制造园等58个内资项目，积极谋划建设“中日循环经济产业园区”和“中韩物流园区”。全年利用外资9.5亿美元。努力扩大对外贸易。坚持出口市场多元化、集约化，以质取胜，用好国家出口退税政策，调整优化出口产品结构，发展壮大陶瓷、钢材、机械制造、农产品等外贸出口基地，鼓励企业积极抢占国际市场。围绕产业发展需要，积极支持关键设备、先进技术和短缺资源进口。支持有条件、有实力的企业“走出去”，更好地利用两个市场、两种资源。全市进出口总额增长11%。积极推进区域合作。全方位参与环渤海地区和京津冀都市圈产业分工与对接，尤其是加强与京津地区的战略合作。认真落实省委、省政府打造冀东经济区部署，支持曹妃甸新区承德临港工业园和秦皇岛临港工业园建设，促进唐秦承区域经济一体化发展。

(八)以经济社会协调发展为目标，全面发展各项社会事业。在发展经济的同时，更加重视社会建设，不断加大社会事业投入力度。今年全市财政投入社会事业资金增长15%以上。

优先发展教育事业。围绕实现教育公平，整合教育资源，优化教育布局，加大教育投入，健全义务教育经费保障机制，促进各类教育均衡发展。巩固提高九年义务教育发展水平，加强中小学生思想道德教育，积极推进和深化开放式素质教育试验，继续推进普通高中免费教育。坚持以就业为导向，大力发展职业教育，积极创建国家级、省级示范校和示范性实训基地，加快建立以服务唐山经济社会发展为核心的职业教育体系。加快发展高等教育，加强高校重点学科建设，积极推进与国内外知名高校的交流与合作，全力支持河北理工大学和华北煤炭医学院争取博士学位授予权，启动曹妃甸科教城建设。完善成人教育体系，积极发展民办教育。加强教师队伍培训，提高教师队伍素质。

大力推进文化建设。深入推进文化繁荣工程，大力发展文化事业和文化产业。加快青少年宫、科技馆等重点项目建设，启动文化艺术广场、传媒大厦、中国近代工业博物馆、档案馆、动漫文化主题公园、文化创意产业基地等文化基础设施和文化产业项目建设。大力培养文化艺术人才，组织推出一批优秀剧目和作品，办好社区文化艺术节、高雅艺术下基层等系列群众文化活动。以纪念评剧诞生100周年为契机，进一步弘扬冀东优秀地域文化。深入开展文明城市创建活动，大力弘扬新唐山人文精神，强化公民道德教育和实践，不断提升全市人民思想道德水准和精神境界，促进人的全面发展。

加强医疗卫生事业。健全完善城乡卫生服务体系，提高基层医疗卫生服务水平。加快市工人医院外科大楼、市人民医院肿瘤大楼建设，启动市中医院扩建、市儿童医院项目，改扩建市传染病医院，谋划建设新工人医院、口腔医院和精神病院。加强传染病预防控制，提高突发公共事件应急处置能力。强化卫生执法监督，进一步规范药品生产流通秩序，推进医疗垃圾无害化处理，确保人民群众就医用药安全。按照国家统一部署，稳步推进医药卫生体制改革。

加快发展体育等各项事业。广泛开展全民健身运动，实施奥运人才培养工程，提高竞技体育水平。谋划启动新奥林匹克体育运动中心建设。实施计生惠民工程，稳定低生育水平，提高人口素质。加快广播电视数字化发展。继续做好国防动员、人民防空工作，加强民兵预备役建设，深入开展双拥共建活动。重视发展民族宗教、外事侨务、气象、地震、档案、老龄、妇女儿童、残疾人等各项事业，促进社会全面进步。

(九)以持续改善民生攻坚行动为重点，进一步提高人民群众幸福指数。建设科学发展示范区，最终目的就是让全市人民生活得更加幸福。全面落实“十大幸福工程”，实施持续改善民生攻坚行动，切实解决好群众最关心、最直接、最现实的利益问题，把发展为了人民、发展依靠人民、发展成果由人民共享落实到改善民生的一件件实实在在的事情上。

全年计划投入60亿元，为群众办好20件实事。围绕扩大就业，全面落实就业创业各项促进扶持政策，以创业带动就业，努力增加就业岗位，突出做好下岗失业人员、就业困难群体、零就业家庭、高校毕业生、退伍军人就业安置工作。全年城镇新增就业6.9万人，安置就业困难群体5000人以上，实现“零就业”家庭动态归零。围绕劳有所得，建立健全企事业单位工资正常增长机制,努力增加中低收入群体收入。妥善解决好拖欠农民工工资问题，依法维护劳动者权益。围绕住有所居，完善住房保障制度，全市开工经济适用住房61万平方米、廉租房25万平方米，其中市中心区建设经济适用住房40万平方米、廉租房10万平方米，全部解决城市住房困难群众的住房问题;对1000户农村贫困残疾户危房进行改造援助。围绕老有所养，按照国家、省部署继续提高企业退休人员养老金水平，加快推进农村新型养老保险;进一步提高城乡低保标准，城镇由每人每月270元提高到285元，农村由每人每年1200元提高到1300元。围绕病有所医，全面推进城镇居民基本医疗保险，将高校在校生、城中村人员、外来务工人员家属子女纳入城镇居民基本医疗保险参保范围，建立居民大额补充医疗保险制度，提高保障水平;巩固和发展新型农村合作医疗制度，加强对贫困残疾人员的医疗救助。围绕全民健康，启动“健康唐山、幸福人民”行动，实施健康教育、健康服务、健康饮食、健康文体、健康环境“五大工程”，为全市每位公民建立一个健康档案，制订一个健康计划，力争每年进行一次健康体检，使全市人民主要健康指标实现明显提高，健康环境得到明显改善。围绕改善环境质量，深化钢铁、焦化、电力、水泥等重点企业环保达标建设，对10家重点行业企业进行脱硫改造，综合整治燃煤锅炉;完成市中心区民用天然气置换;全部消灭城市裸露地，治理工地、公路和街道粉尘污染，减少粉尘对人体危害。城市环境空气质量二级及优于二级天数达到310天以上，可吸入颗粒物、二氧化氮年均浓度值稳定达到国家二级标准。围绕方便群众出行，新建、改建农村公路700公里，改造农村公路危桥1500延长米;打通4条城市断头路，新建外环线连接道路2条，启动建设2座城市高架桥和2座立交桥;新购置公交车60部;实行城市65岁以上老人免费乘坐公交车。围绕便民服务，完善560个村民中心,建设500个规范化村民中心，全市所有的村建成村民中心;加强城市社区建设，建设100个社区市民中心。推进家电下乡工程，完善销售网络，及时对农民购买的彩电、冰箱、洗衣机、手机等四类家电下乡产品按国家政策给予补贴。

(十)以安全生产和食品药品安全整治攻坚行动为突破口，努力维护公共安全和社会和谐稳定。安全发展直接关系人民群众切身利益和生命财产安全。牢固树立安全发展理念，坚持把安全生产和食品药品安全整治工作作为“天字号”工程,摆在科学发展示范区建设的突出位置，下决心、下大力抓紧抓好。

在安全生产方面，汲取安全生产事故的深刻教训，强化安全生产责任和各项措施的落实，坚决遏制重特大事故发生。一是完善安全生产工作领导机制。严格落实各级党政领导干部“一岗双责”责任制，进一步强化各级政府主要领导安全生产第一责任人、主管领导直接责任人的责任，切实加强对安全生产工作的领导，形成安全生产工作新格局。二是进一步明确各级安全监管部门的责任，加强对各级各类企业的安全生产监管。实行“谁检查、谁签字、谁负责”的责任制和问责制。三是强化企业安全生产主体责任。严格落实相关政策措施，用法律和政策把安全生产主体责任落实到每个企业。加大企业安全生产投入，改善安全生产条件，落实安全生产措施，健全规章制度，提高企业安全生产整体水平。在钢铁、煤矿等重点行业全部安装数字化报警系统。四是坚持不懈开展安全生产大检查。特别是对煤矿、非煤矿山、冶金企业、危险化学品、人员密集公共场所、易燃易爆单位、建筑施工企业等高危行业和重点领域持续实施安全生产专项整治，并建立经常性的排查监管机制，彻底消除事故隐患。五是加强安全培训。建立完善市县两级安全生产培训中心和基地，全面落实企业安全生产培训保证金制度，对所有企业员工开展安全生产培训。六是严格安全生产执法和责任追究。严厉打击各种安全生产非法行为，加大责任追究力度。对因安全责任不落实、措施不到位、监管不力引发安全生产事故的政府工作人员和企业法人(控制人)坚决按照“四不放过”原则，依法追究责任、严肃处理，决不姑息迁就。

在食品药品安全方面，建立健全联合执法和监管体制，完善检验检测体系，对种植养殖、生产加工、包装、储运、销售等各个环节进行严密监管，实施全流程监测;加强执法检查，从重从快严厉打击涉嫌生产、销售有毒有害及假冒伪劣食品药品的行为，发现一起、查处一起;发展生态农业，努力增加优质安全食品供应，让群众吃上放心菜、放心肉、放心粮;广泛发动群众监督，建立举报奖励制度，在全市营造人人关心、支持食品药品安全的社会氛围。

在维护社会稳定方面，全面实施维护稳定综合调控，建立健全多元化的社会矛盾纠纷化解机制，正确处理新形势下的人民内部矛盾，切实做到“小事不出村(居)、一般事不出乡镇(街道)、大事不出县区、矛盾不上交”。高度重视和切实加强社会治安综合治理，深入推进新一轮“诚信平安唐山”创建活动，健全完善社会治安防控体系，进一步提高对社会治安秩序的控制能力，严密防范和依法严厉打击违法犯罪活动。强化全民普法教育，不断提高全民的法律道德素质。继续开展好市、县、乡、村四级大接访，进一步规范信访秩序，深入推行信访代理制，带着感情做好群众工作。完善应急预案体系，妥善处置群体性事件，切实提高预防和处置突发公共安全事件的能力。

四、切实加强政府自身建设

加快建设科学发展示范区、建设人民群众的幸福之都，各级政府责任重大、使命光荣。我们必须带头落实省委和市委关于开展“作风建设年”工作部署，进一步加强政府自身建设，努力开创政府工作新局面。

(一)不断解放思想，提高创新力。巩固深化科学发展观学习实践活动成果，更新理念，创新思维，自觉运用党的最新理论成果武装头脑，自觉摒弃不合时宜的传统观念和思维定势，切实把思想和行动统一到中央、省和市委决策部署上来。面对新形势、新任务，以敢为人先、勇于探索的精神，研究新问题，探索新规律，制定新举措，实现新跨越。加强公务员队伍建设，有计划开展公务员培训，引导各级公务员学习新知识、练就新本领，增强服务发展、服务人民群众的能力。

(二)加强效能建设，提高履职力。针对不作为、乱作为问题，动实招、硬奖惩，切实加强政府机关效能建设，努力提速工作过程，提高行政效能。结合开展“作风建设年”活动，对工作目标实行项目化管理，解决不作为问题;对工作过程实行标准化管理，解决乱作为问题;对工作效率实行档案化管理，解决无效作为问题。进一步创新行政管理体制，全面落实首办责任制、限时办结制、超时默许制和联合审批制，整合审批流程，公开审批程序，提高审批效率，降低行政成本，提升服务水平，优化发展环境。严格实施政府机关干部综合考评，考核结果向社会公开，接受群众监督，实行末位淘汰和问责制，切实做到权责统一、责任明确、严格考核、奖罚分明。

(三)坚持真抓实干，提高执行力。大力弘扬求实精神和务实作风，少说多做，力戒浮躁，坚决反对形式主义、官僚主义，扎实推进工作落实。以时不我待、奋发有为的精神状态抓落实，强化机遇意识、责任意识、效率意识，拼搏进取，加压奋进;坚持突破重点、活跃全局抓落实，统筹兼顾，抓重点、攻难点，尤其对事关全市科学发展全局的战略重点和群众关心关注的热点难点问题，集中力量实施攻坚突破;全面推行“一线工作法”抓落实，从市政府各部门做起，深入基层掌握实情、破解难题、搞好服务，真正做到干部在一线工作，决策在一线落实，问题在一线解决，创新在一线体现，成效在一线检验。

(四)推进依法行政，提高公信力。自觉接受人大法律监督、政协民主监督和社会监督，完善和加强政府内部监督，确保行政权力依法公正行使。深入推进行政权力公开透明运行，自觉接受群众监督。健全社情民意反映制度和重大事项专家咨询、社会公示、听证和质询制度，继续开展“市民献计献策月”活动，切实做到科学决策、民主决策。继续深入开展民主评议行风政风活动。坚持依法行政，推动政府各项工作法制化。

(五)保持清正廉洁，提高感召力。加强对权力运行的监督和制约，更加注重治本、更加注重预防、更加注重制度建设。坚持不懈开展反腐倡廉教育，筑牢拒腐防变的思想道德防线。认真坚持党风廉政建设责任制，健全和落实“一岗双责”制度。强化对行政权力运行的监督和制约，完善政府投资监管、国有资产监管和非经营性资产管理制度，规范建设工程招投标、经营性土地使用权出让、政府采购和产权交易制度，用制度管权、管事、管人。加大专项治理力度，解决好土地征用、房屋拆迁、环保等方面群众反映强烈的问题，坚决纠正损害群众利益的不正之风。坚持从严治政，廉洁从政，努力在人民群众中树立良好形象。

各位代表，我们深知人民政府的权力是人民赋予的，政府的职责就是为人民服务。我们一定牢固树立正确的政绩观，强化民本意识，深怀爱民之心，恪守为民之责，善谋为民之策，多办利民之事，真正做到感情上贴近群众、工作上深入群众、发展上依靠群众，最大限度地实现好、维护好、发展好广大人民群众的根本利益，以实际行动造福全市人民。

各位代表，今年的形势复杂而严峻，任务光荣而艰巨。让我们更加紧密地团结在以胡锦涛为总书记的党中央周围，在省委省政府和市委正确领导下，全面贯彻落实科学发展观，发扬求真务实、真抓实干的作风，开拓创新，拼搏进取，团结奋斗，全面完成今年确定的各项目标任务，以建设科学发展示范区和人民群众幸福之都的优异成绩向建国六十周年献礼!
